# Financing Agriculture in Nigeria through Agricultural Extension Services of Agricultural Development Programmes (ADPs)

**DOI:** 10.12688/f1000research.16568.3

**Published:** 2019-05-30

**Authors:** Henry Inegbedion, Eseosa Obadiaru, Barnabas Obasaju, Abiola Asaleye, Adedoyin Lawal

**Affiliations:** 1 Department of Business Studies, Landmark University, Omu Aran, Kwara, +234, Nigeria; 2 Department of Accounting and Finance, Landmark University, Omu Aran, Kwara, +234, Nigeria; 3 Department of Economics, Landmark University, Omu Aran, Kwara, +234, Nigeria; 4 Department of Banking and Finance, Landmark University, Omu Aran, Lwara, +234, Nigeria

**Keywords:** Agricultural Extension Services, ADP, Agricultural development

## Abstract

The ADPs were designed in response to a fall in agricultural productivity and hence a concern to sustain domestic food supplies. The study examined “Financing Agriculture in Nigeria through Agricultural Extension Services of Agricultural Development Programmes.” It sought to ascertain the extent to which agricultural extension services of the agricultural development programmes have impacted the financing of agriculture in six selected local government areas in Edo South senatorial district, Nigeria using a sample of 120 respondents. Stratified random sampling was used to select the respondents. Interview schedule served as the research instrument. The research data were analyzed using t-test and Pearson correlation, which served as the inferential statistics. The research findings showed that the extension services of ADP have impacted significantly on crop development in the selected communities but have not had significant impact on employment creation and the development of infrastructural facilities. The study also revealed that there was no significant difference between the implementation of the projects in the selected communities, as revealed by the correlation test. On the basis of the research findings, the need for a complete redesign of the project to ensure that it achieves its stated goals as well as ensure proper monitoring of its implementation were suggested, among others.

## Introduction

Prior to independence, agricultural production was the mainstay of the Nigerian economy. Even following independence the scenario did not show any change as agriculture contributed well over 90% to the nation’ foreign exchange earnings. However, with the discovery, exploitation, and exportation of crude oil in commercial quantities, the contribution of agriculture to the nation’s foreign exchange and GDP began to dwindle as attention shifted from agriculture to oil ( Inegbedion, 2012). The domination of the economy by the oil sector caused the Nigerian economy to become a monoculture, with the attendant implications. This led to the disengagement of many able-bodied men from productive activities in search for oil money, thus precipitating an unprecedented rural-urban drift in the 1970s. The neglect of the agricultural sector and the subsequent rural-urban drift created a visible dent in Nigeria’s food supply, and thus signaled the need to embark on massive importation of food stuffs in the early 1980s.

The government’s response to the glaring distortion was to embark on a series of interventionist programmes referred to as the Agricultural Development Projects (ADPs). The ADPs were designed to increase crop production with the major components being improving technology, increasing the supply of farming and crop inputs, as well as reasonable improvements in basic infrastructure ( Ammani *et al.*, 2010). The ADP was kick started with three pilot programmes in the northern part of the country. The perceived success of the pilot programmes necessitated a clarion call for its expansion ( Independent Evaluation Group, 2009: 1). This led to the replication of the projects from the early 1980s all through the decade. Bendel ADP, which later became Edo ADP in 1992, was among this group. Each of these groups included four basic components:- farm and crop development (expanded research, extension, and input supply), infrastructure development (feeder road construction and maintenance, water supplies, markets and storage facilities), institutional support, establishment of project entities separate from the state agriculture departments, and technical assistance, largely to manage the new institutions. Owing to the perceived importance of ADP to agricultural development in Nigeria several studies have been conducted to evaluate its performance since inception, some of the recent studies include and Adamu & Mohammed (2009); Ammani *et al*. (2010); Enwelu *et al*. (2017); Naswem & Ejembi (2017); Nchuchuwe & Adejuwon (2012); Omonijo *et al*. (2014); Ugwu (2007); Umeh *et al*. (2015), as well as Olujenyo (2006), among others.

### Objectives of the study

The study sought, mainly, to investigate the impact of agricultural extension services of Edo state ADP on agricultural financing in Edo state using six selected communities in three local government areas in Edo south senatorial district, by focusing on the activities of Edo ADP Extension Division. The specific objectives of the study were to determine: The extent to which the extension services of Edo ADP have impacted farm and crop development in the selected communities under study; the extent to which the extension services of Edo ADP have impacted infrastructural development in the communities under its coverage, and the extent to which the extension services of Edo ADP have contributed to the reduction of unemployment through the attraction of able-bodied men and women to agriculture.

## Literature review

There are numerous accounts of some countries that use to be buoyant but later nosedived, due partly to mismanagement of environmental resources. Learning from such accounts it is imperative that policy makers be conscious on the need for effective management of their environments with a view to minimizing the cost of producing natural products like food, fibers, and associated resources, while mindful of the need to minimize the risk of to the survival of future generations ( Tunji Titilola, 2001) As a major component of economic development, the ADP was meant to boost production and productivity for some reasonable period of time as well as the wellbeing of farmers; which was supposed to translate to a higher per capital income of the economy. Rural development, which is part of the central focus of agricultural productivity, is not only concerned with a sustainable increase in the levels of productivity and production of farmers and other rural dwellers; neither is it only concerned with a significant enhancement of the wellbeing of rural dwellers as reflected in increased per capita income and standard of living, but it is also expected to translate to a substantial and sustainable enhancement in the social and economic wellbeing of the rural communities ( Nchuchuwe & Adejuwon, 2012). Rural development can thus be seen as instrumental in combating deprivation and poverty in order to enhance economic prosperity at the grassroots. From the point of view of most countries, rural development refers to a sustainable increase in the productivity and earnings of households and low income workers from rural areas ( Nwachukwu & Eze, 2007).

### Concept of ADP

Dwindling agricultural productivity in the mid-70s necessitated the establishment of the ADPs; the goal was to sustain domestic food supplies in the country given the migration of labour from agriculture to other sectors that were perceived to be more lucrative at the time, especially in the cities, owing to the activities from the oil boom ( Independent Evaluation Group, 2009. Available from the World Bank Group website). On the other hand, government was afforded the resources and opportunity to develop the ADPs by the domestic recycling of oil income. The ADPs, consistent with their purpose, provided opportunity for investment in agriculture and agricultural services, and infrastructural facilities such as access roads and pipe borne water in the rural areas. The establishment and adoption of the ADPs by government situated the smallholder sector at the center of its agricultural development strategy; this action marked a conspicuous shift from the previous capital intensive investment projects targeted at selected areas designated as having high agricultural potential.

The pioneer ADPs were community projects with each encompassing specific regions within a state. The results of the implementations were impressive to the policy makers at the federal, state and local governments. To this end, the governments were pressured to replicate the projects in all the remaining communities across all the states of the federation. As a result of the pressure and expectations, all 19 states of the federation had ADPs by 1989. The major goal was to increase food production and farm incomes in all host communities across the federation. The thinking was that increases in production and productivity would be a direct consequence of improved technology, especially planting material and fertilizer. The design of the agricultural components of the ADPs was centered on systems for the development and transfer of technology to farmers, and the distribution of modern inputs and land development. These included land clearing and small scale irrigation of irrigable areas in the northern parts of the country. Investments in infrastructural component of the programme included the construction of expanded feeder road network, construction of farm service centers for the distribution of crop and farm inputs, as well as the provision of facilities for the operations of ADP staff. With the exception of the Ilorin ADP, all the other projects across the country supported improvements in rural water supplies.

***Edo state ADP*.** ADP was established in Benin City in 1986 with focus on all the local government areas in the old Bendel state. With the creation of Edo state in 1991, the focus (coverage areas) of the scheme became restricted to all the local government areas in Edo state. The scheme is a tripartite arrangement between the federal, states, and local governments just like the programmes in northern Nigeria. That notwithstanding, at the moment, the state government is solely responsible for the payment of staff salaries. The areas of intervention of Edo ADP include; infrastructural development, such as construction and maintenance of earth roads,; provision of water through the sinking of Boreholes in the communities,; building of markets,; and provision of storage facilities,; as well as farm and crop development (rural agriculture) through the provision of fertilizers, pesticides and farm implements to rural farmers at subsidized rates as well as the provision of consultancy services and sensitization of farmers.

**Figure 1.**
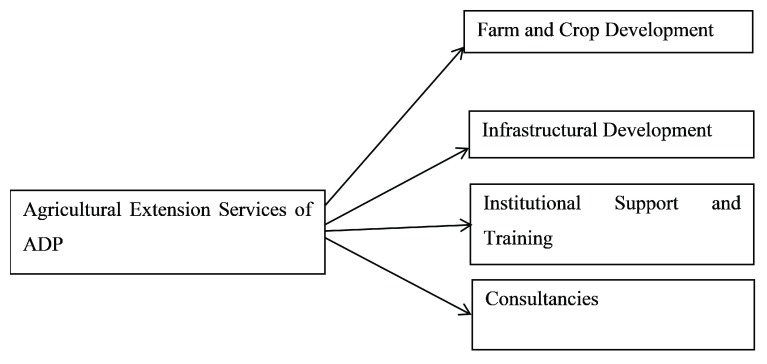
Conceptual model (Authors’ proposition, 2017).

The FADAMA programme of the ADP, which began in the north has also taken off in the south, and is currently undertaken by Edo ADP. FADAMA means irrigable, and it involves interventionist programmes using relevant methodologies in areas where water is close to the surface of the earth. The frequency of intervention is contingent upon necessity as well as availability of funds, but also subject to political consideration at times. The ADP mandate includes farm and crop development, infrastructural development, institutional support and training, as well as consultancies. The major constraints currently being faced by Edo ADP include: Inadequate manpower, and the preference of the state ministry of agriculture over ADP by the administration of Edo state. The situation was alleged to be threatening the capacity of the Edo ADP to embark on its traditional interventionist programmes since such functions are now being contracted to the Ministry of Agriculture as of mid-2000. A framework of the study is presented (see Figure 1).

### Empirical review

Naswem & Ejembi (2017) investigated “reviving agricultural extension for effective transition from subsistence to commercial agriculture”. The purpose of their study was to identify the factors responsible for the erosion of the extension system, and identify a reliable path that will make the system come alive again. This was to trigger the new transformation agenda policy in agriculture. They highlighted the weaknesses of past extension efforts. The need for the younger generation to be deliberately involved in agriculture was suggested, among other recommendations.

Auta & Dafwang (2010) investigated the status and policy of ADPs in Nigeria. They found that over 63% of the ADPs had a weak or very weak funding status while over 22% had a good to excellent status. Furthermore, most of the ADPs had reduced their extension workers in recent times due to poor funding.

Chukwuemeka & Nzewi (2011) investigated World Bank sponsored Agricultural Development Project to find out the extent to which the programme had achieved set objectives. Survey and questionnaire served as research design and instrument. Findings showed that beneficiaries were excluded from design, planning and implementation of project; a development that was perceived as undesirable. Besides, political considerations, rather than expertise and professionalism was found to characterise the recruitment of extension staff; and the joint financiers (World Bank, Federal and State governments of Nigeria) were not fulfilling their financial obligations as at when due.

Okuokenye & Okoedo-Okojie (2014) investigated activities of extension agents on agricultural loans and inputs supply programme of participant farmers’ rice output/income. Questionnaire served as the instrument of data collection from a sample of 60 randomly selected extension farmers and 80 participant and 80 non-participant farmers’. Results showed that the extension agents made significant impact on output and income. The major constraints to the implementation of the programme were found to be restricted coverage of farms and wrong selection of participants

Ammani *et al*. (2010) investigated the “challenges to the sustainability of the ADP system in Nigeria”. The purpose of their study was to analyse the problems perceived to be constraining the sustainability of the ADP, and as a consequence, the effective performance of the ADP system in Nigeria. Inadequate funding was viewed as the focal problem. They developed and transposed a problem tree and used it to transform the identified root causes, and consequences into root solutions. Based on their findings, they suggested that government should focus on improving funding for the ADPs, making deductions from state and federal government revenue allocations from source through a counter-part funding arrangement for the ADPs.

Olujenyo (2006) investigated the “impact of ADP on the quality of social existence of rural dwellers in developing economies in Ondo state (Nigeria)”. The purpose of the study was to examine the extent to which the implementation of the ADP had impacted the rural farmers in Ondo state of Nigeria, West Africa through an investigation of the impact of the programme on the farming operations of the target farmers, as well as the organizational, and farm-related factors perceived to be associated with the impact of the ADP. A survey design was employed and structured questionnaires served as the research instrument. The research instrument was used to ascertain the perception of 288 respondents about the performance of the ADP programme in terms of its impact on the rural farmers. The respondents consisted of 144 contact farmers and 144 non-contact farmers. Contact farmers are those who belong to cooperative societies, while non-contact farmers do not belong. Random and systematic sampling served as the sampling techniques. Research data were analysed using correlation technique. Following the inferential analysis of the data, it was found that average yields per hectare of land cultivated by the farmers differed significantly from the average score of the articles of convenience owned by the farmers before implementation and after the implementation of the ADP in all the four crops examined.

Nchuchuwe & Adejuwon (2012) sought to investigate the constraints to agricultural and rural development in Nigeria. They observe that agricultural activities in Africa were still largely traditional and mainly concentrated in the hands of pastoralists and smallholders and that the relegation of the agricultural sector has precipitated negative net migration. They further discuss the consequences occasioned by the problems and challenges resulting from the neglect of the agricultural sector, and government responses to the rural infrastructural needs of the people. Among other needs, they suggested the provision of an adequate level of strategically targeted investment in agriculture as well as the upgrade of rural infrastructure; concerted efforts at boosting of productivity and increased competitiveness of the farm output.

Ugwu (2007) examined “contributions of ADPs to rural livelihood and food security in Nigeria” He explored the programme from its inception in order to give a valuable account of its impact. It was observed that the primary purpose of establishing the ADPs was to stimulate agricultural production and thus help to bring about the enhancement of the wellbeing of the rural dwellers and provision of adequate food in all the benefiting communities in particular and the entire country in general. He identified the contributions of the ADPs to be mainly in the resuscitation of the extension service, enhancement of the knowledge of the local farmers, provision of basic infrastructure in the rural areas, provision of agricultural input to farmers,; development, transfer and adoption of adequate technology, as well as enhancement of the livelihood of individuals in the rural areas, and provision of adequate food as well. Furthermore, they observed that the continuity of the programme was guaranteed by the conspicuous successes of ADP in the target areas. However, political interference which are often unwarranted, unstable inflow of the required money due mainly to default in the payment of counterpart funding by the three tiers of government, rapid staff turn-over in most ADPs, among other factors, were identified as the major constraints to the success of the programme. The need for government to give increased political support was suggested.

Adamu & Mohammed (2009) investigated “the effect of ADP on the rural farmers in Adamawa state, Nigeria”. The authors collected data on annual crop output, income, farm size, and the availability of improved technology, access to credit and training of farmers and rural infrastructure using a structured questionnaire and personal interviews,; t tests were used to analyze the data. The results indicated a positive, and significant impact of the ADP in Adamawa state on the productivity, income, access to credit, and standard of living of rural farmers using assets ownership criterion. The study did not reveal any significant impact of the ADP on the adoption of improved technologies, rural infrastructure, and farm sizes. Consequently, the need to enhance the provision of rural infrastructure, and technologies; as well as fund the project adequately was recommended, among others.

Omonijo *et al*. (2014) examined “impacts of ADP on rural dwellers in Nigeria using the people of Isan Ekiti, Oye Local Government Area of Ekiti state as case”. A survey method was employed and a questionnaire served as the instrument. They retrieved and analysed 73 questionnaires using descriptive and inferential statistics. Multiple linear regression analysis served as the inferential test. Results reveal that there was a significant relationship between ADP (through increased provision of pesticides, improved seeds to farmers, establishment of new infrastructure, as well as provision of fertilizers) and increased food production in the locality. However, accessibility of credit by farmers had no significant effect on increased agricultural productivity. The need for government to increase its effort in the area of agricultural credit financing was suggested.

Another investigative study was conducted by Umeh *et al*. (2015). The study compared the performance of the ADP of Abia with that of Enugu states in Nigeria. The authors evaluated the performance of the programmes in the two states with particular focus on agricultural extension delivery services. They used multi-stage sampling to select 200 respondents. Primary and secondary data were employed. The paired t-test was used in hypothesis testing. Results showed that three out of the 11 performance indices of the two States were significantly different at the 95% level of confidence. Consequently, the need for government to expedite action in the employment of better trained extension staff to facilitate the enhancement of service delivery in Enugu State was suggested.

Enwelu *et al*. (2017) investigated the “access and use of information communication technologies in Anambra State ADP”. A sample of 69 respondents was selected and investigated while a structured questionnaire served as the research instrument that was used to elicit the desired data. The data were analysed using descriptive and inferential statistics. The inferential statistics consisted of factor and regression analysis. The extension workers and workers in ADP office had high levels of access to mobile phones, and used phones very often. The major constraints to the access and use of other ICT facilities were found to be soaring cost of maintenance of ICT tools, dearth of competence in utilization of ICT and inadequate support by organization and government, among others.

***Research Gap.*** The empirical review reveals that adequate empirical literature abound on agricultural financing through the extension services of ADP in Nigeria. Most of the studies agree that the ADPs have made significant impact on agricultural production in Nigeria, especially in the a areas of increased agricultural output and income as well as improved rural livelihood ( Adamu & Mohammed, 2009; Ammani *et al.*, 2010; Okuokenye & Okoedo-Okojie, 2014; Olujenyo, 2006; Omonijo *et al.*, 2014; and Ugwu, 2007). However, not all the objectives of the programme have been successful. Specifically, the provision of credit facilities ( Omonijo *et al.* (2014) and infrastructural developments ( Adamu & Mohammed, 2009). Furthermore, despite the perceived positive impact of the ADP in agricultural outputs and income, findings also indicate that there are challenges currently being faced by the programme in a significant number of the states where it is being implemented. These challenges could erode the credibility and “Goings Concern” of the implementation of the project if urgent steps are not taken to mitigate the challenges. The major challenges include inadequate funding, mainly as a result of the inability of critical stakeholders (World Bank, federal government and state governments) to fulfill their financial obligations to the programme as and when due ( Ammani *et al.*, 2010; Chukwuemeka & Nzewi, 2011; Ugwu, 2007) Omonijo *et al.*, 2014; Auta & Dafwang, 2010; and Okuokenye & Okoedo-Okojie, 2014) as well as politicization of the selection of participants in the implementation of the programme by government ( Chukwuemeka & Nzewi, 2011; Okuokenye & Okoedo-Okojie, 2014; and Ugwu, 2007).

In view of the foregoing, the need to re-awaken the interest of stakeholders on the actualization of the objectives of the ADP becomes very important. Besides, the perceived insignificant impact on infrastructural development should attract the interest for stakeholders for adequate intervention. The constraints to the continued implementation of the scheme also raise some questions to stakeholders. This study attempts to fill these gaps

## Methods

The study adopted conclusive research design in order to determine the extent to which the Agricultural Extension Services influences community development in Edo State. This is consistent with Inegbedion & Obadiaru (2018). The population of the study consisted of small scale farmers in Edo South senatorial district since they were the group to whom the generalization of the results of the study was intended ( Agbonifoh & Yomere, 2002). Six communities, two each from three local government areas in the old Oredo local government area – Ikpoba-Okha, Egor, and Oredo local government areas were the focus of the study. The communities were Iyekogba, Ogba, Etete, Evboriaria, Egor and Utoka. The study was conducted between December 2012 and January 2013.

The sample size was computed using the formula $\frac{z^{\text{2}}p(1-p)}{e},.$ Here, n represents the sample size, Z is the score corresponding to the level of significance and representing the abscissa on a normal curve in area α to minus infinity at the tails, while 1 – α is the confidence level corresponding to the level of significance, α , e.g., 95% for α = 0.05; e is the desired level of precision, p is the estimated proportion of farmers in the population and q is 1-p. The value for Z is obtained from a normal distribution table, based on the area under the normal curve ( Cochran, 1963; and Agbadudu, 2007).

The aim was to be 95% confident that the perception of the sampled respondents will not differ by more than 5% from the perception of the true population of the study. Furthermore, approximately 40% of the people in the communities are farmers. Consequently, $\text{n}=\frac{z^{\text{2}}x\;\;0.4\;x\;\;\text{0}.6}{\text{0}{\text{.05}}^{\text{2}}}$ = 368.8. This value was approximated to 366, hence a sample size of 366 was used. Given the sample size of 366, 61 respondents were scheduled to be sampled in each community. The study was done in the communities but the community centers around the village heads served as the take-off points in each of the communities. The communities were stratified according to the nearest 50 compounds to the residence of the village head. Subsequently, simple random sampling was used to select the desired number of respondents from each community. Thus, the sampling technique used was stratified random. An interview schedule was then used to elicit the necessary information from the sampled respondents. The choice of the interview schedule as the research instrument was informed by its flexibility which allows items to be adapted to the respondents’ level of education. The study was conducted between December 2012 and January 2013 in Edo state of South-South Nigeria by this author and a research assistant who was paid to assist in conducting the interview. The interview schedule had two parts – the Bio-data, which featured items on the respondents’ demographic characteristics and the core-subject matter, which featured items that addressed the research problem. The question-response format of the research questions was the Likert type five point scale with options ranging from a region of strong agreement (strongly Agree), through a neutral zone (Not Sure), to a region of strong disagreement (Strongly Disagree). As it is common with all likert-scale items, the questions sought to ascertain respondents’ perception of the research problem. The questionnaire used is available as appendix in this paper.

Research data were analyzed, using descriptive statistics such as mean, standard deviation, mean difference and standard error mean; as well as one sample t test and F ratio test (ANOVA) and regression analysis, which served as inferential statistics. The one sample t test was used to test for significance of the independent variables while regression was used to examine their predictive power. ANOVA was performed to determine the existence of differences in in ADP across locations. This is consistent with Inegbedion *et al*. (2016). Data analysis implemented using the Statistical Package for Social Sciences (SPSS) 20.0. The study was reported using the SRQR reporting guidelines.

## Ethical considerations and consent

The study was an Assignment which formed part of the course work in Economic Analysis, in partial fulfillment of the requirements for the award of a Doctor of Philosophy (Ph. D) in Business Administration of the University of Benin, Nigeria. The ethics committee indicated no ethical approval was required for this study.

Co-authors were included to assist in enhancing the quality of the work. Verbal consent of the respondents was sought after assuring them of their anonymity. The respondents thus gave their consent willingly as they fully understood the purpose of the study. Due to time restrictions and the low risk nature of the study, verbal and not written informed consent was obtained from participants.

## Results

### Introduction

Out of the 366 respondents scheduled to be sampled, information was elicited from 248 of them. The age distribution of the respondents shows that 12 were under 30 years, 75 were in the age group 31–40 years, 105 were in the age group 41–50 years and 56 were above 50 years, thus indicating that majority of them were in the age group 41–50 years. The sex distribution shows that 169 of them were male while 79 of them were female, thus indicating that majority of the respondents were male. The distribution by marital status shows that 191 of them are married, 38 are single, 15 are widowed, while 4 are divorced; thus showing that majority of them are married. The distribution by highest educational level showed that 113 of them have Secondary School Certificate (SSCE), 82 had National Diploma or National Certificate Examination Certificate (ND/NCE); lastly, the distribution of the respondents by local government showed that 82 of them were from Oredo local government, 90 from Egor local government and 76 from Ikpoba Okha local government; thus majority of the respondents were from Egor local government area (see Table 1). The summary of the respondents’ perception is presented in Table 2. The results of the data analysis are presented in Table 3– Table 8.

**Table 1.** Demographic Distribution of respondents.

Age	Category	F	%
	Under 30 Years 31–40 Years 41–50 Years 50 Years and above Total	12 75 105 56 248	4.8 30.2 42.3 22.6 100
Sex	Male Female Total	169 31.9 248	68.1 31.9 100
Marital Status	Married Single Widowed Divorced Total	191 38 15 4 248	77 15.3 6 1.6 100
Highest Education	SSCE ND/NCE HND/First Degree Higher Degree Total	113 82 51 2 248	45.6 33.1 20.6 8 100
Local Govt. Area	Oredo Egor Ikpoba Okha Total	82 90 76 248	33.1 36.3 30.6 100

**Table 2.** Response to Items on Research Problem.

Variable	Item	SD	D	NV	A	SA
1. Farm and Crop Development	Q1 Q2 Q3	10 14 9	70 75 72	58 54 61	62 79 81	28 26 25
2. Infrastructural Development	Q4 Q5 Q6	20 14 21	76 73 71	53 57 57	75 79 70	24 24 29
3. Reduction in Unemployment	Q7 Q8	17 18	75 24	55 57	78 78	23 21

**Table 3.** Agricultural Extension Services of Edo Agricultural Development Programme (ADP) and crop development.

Responses	N	Mean	Standard Deviation		Standard Error Mean	
One Sample Test	248	3.1694	1.05494		0.06699	
	Test value = 3					
					95% confidence interval Of the difference	
	t.	df	Sig.2-tail	Mean Difference	Upper	Lower
Responses	2.528	247	0.012	0.1694	0.0374	0.301

Source: SPSS output

**Table 4.** Agricultural Extension Services of Edo Agricultural Development Programme (ADP) and development of infrastructural facilities.

Responses	N	Mean	Standard Deviation		Standard Error Mean	
One Sample Test	248	3.0618	1.01794		0.06464	
	Test value = 3					
					95% confidence interval Of the difference	
	t.	df	Sig.2-tail	Mean Difference	Upper	Lower
Responses	0.957	247	0.340	0.0619	-0.066	0.189

Source: SPSS output

**Table 5.** Agricultural Extension Services and reduction in unemployment.

Responses	N	Mean	Standard Deviation		Standard Error Mean	
One Sample Test	248	3.0504	1.03701		0.06585	
	Test value = 3					
					95% confidence interval Of the difference	
	t.	df	Sig.2-tail	Mean Difference	Upper	Lower
Responses	0.765	247	0.445	0.0504	-0.079	0.180

Source: SPSS output

**Table 6.** Respondents’ perception and sex.

Independent Samples Test						
		Levene’s Test for equality of variance		t test for Equality of Means		
		F.	Sig.	t	df.	Sig. (2-tailed)
Agric. Ext. Services Vs. Crop Devpt.	Equal variances: Equal V. not assumed	0.21	0.65	-0.86 -0.86	246 154.3	0.393 0.392
Agric. Ext. Services vs. Infrast. Devpt.	Equal variances Equal V. not assumed	0.31	0.58	-0.73 0.72	246 145.4	0.467 0.476
Agric. Ext. Services vs. Reduct. in Unemp.	Equal variances Equal V. not assumed	1.78	0.183	-0.199 0.192	246 140.4	0.842 0.848

Agric. Ext. Services: Agricultural extension services Crop Devpt: Crop development Infrast. Devpt: Infrastructural Development Reduct. in Unemp: Reduction in unemployment

**Table 7.** Agricultural Extension Services of Edo Agricultural Development Programme (ADP) and Farm Development.

	N	Mean	Standard Deviation		Standard Error Mean	
Responses	248	3.1653	1.0689		0.0679	
	Test Value = 3					
					95% confidence interval Of the Difference	
	t.	df	Sig.2 Tailed	Mean Difference	Lower	Upper
Responses	2.437	247	0.016	0.1653	0.0316	0.2990

Agric. Ext. Services: Agricultural extension services

**Table 8.** Extension services of ADP and crop/Farm and infrastructural Development and unemployment Reduction.

Model Summary					
Model	R	R Square	Adjusted E square	Std Error of Estimate	DW- Statistic
	0.729	0.532	0.526	0.957	2.007
Predictors: (crop/farm production, infrastructural development, unemployment reduction)					
Dependent variable: Extension services of ADP					
Model	Sum of Squares	df.	Mean Square	F.	Sig
Regression	252.948	3	84.316	9.2125	0.000
Residual	222.403	243	0.915		
Total	475.352	246			
Dependent variable: Extension services of ADP					
Predictors: (crop/farm production, infrastructural development, unemployment reduction)					
Coefficients					
Model	Unstandardized Coefficients		Standardized Coefficients		
	B	Std. Error	Beta	t	Sig
Constant	-3.216	0.205		-15.67	0.000
Crop/Farm Development	0.421	0.093	0.329	4.518	0.000
Infrastructural Development	0.513	0.115	0.376	4.461	0.000
Reduction in Unemployment	0.103	0.101	0.077	1.018	0.310
Dependent variable: Extension services of ADP					

### Test of hypotheses

Six hypotheses were tested. The results of the tests are presented below

***Agricultural extension services of Edo ADP have not significantly affected crop development in the communities.*** A comparison of Agricultural Extension Services with crop development in the communities revealed that the mean score (using items 1–3) associated with respondents perception of the extent to which Edo ADP has influenced crop development projects in their communities was 3.1694 with a standard deviation of 1.08668 and a standard error mean of 0.0669. When this was compared with a test value of 3, a mean difference of 0.1532 was observed. A t-test for equality of means revealed that this difference was significant at the five per cent level since the significant (2-tailed) probability of 0.027 is less than 0.05, the assumed level of significance. Consequently, at the 95% confidence level, the agricultural extension services of Edo ADP are perceived to have significantly affected crop development in the communities (see Table 3).

***Agricultural extension services of Edo ADP have not significantly affected farm development in the communities.*** A comparison of Agricultural Extension Services of Edo ADP with farm development in the communities revealed that the mean score (using items 4–6) associated with respondents perception of the extent to which Edo ADP has influenced farm development projects in their communities was 3.165 with a standard deviation of 1.0689 and a standard error mean of 0.0679. When this was compared with a test value of 3, it resulted in a mean difference of 0.1653. A t-test for equality of means revealed that the resultant mean difference was significant at the five per cent level since the significant (2-tailed) P Value of 0.016 is less than 0.05, the assumed level of significance. Consequently, at the 95% confidence level, it is safe to conclude that the agricultural extension services of Edo ADP are perceived to have significantly affected farm development in the communities (see Table 7).

***Agricultural extension services of Edo ADP have not significantly affected the development of infrastructural facilities in the communities.*** A comparison of Agricultural Extension Services of Edo ADP with the development of infrastructural facilities in the communities revealed that the mean score (using items 7–8) associated with respondents perception of the extent to which Edo ADP has influenced the development of infrastructural facilities in their communities was 3.0618 with a standard deviation of 1.0179 and a standard error mean of 0.06464. When this was compared with a test value of 3, it resulted in a mean difference of 0.1653. A t-test for equality of means revealed that the resultant mean difference was not significant at the five per cent level since the significant (2-tailed) P-value of 0.340 was not less than 0.05, the assumed level of significance. Consequently, at the 95% confidence level, the agricultural extension services of Edo ADP are perceived to have not significantly influenced the development of infrastructural facilities in the communities (see Table 4).

***Agricultural extension services of Edo ADP have not significantly influenced reduction in the level of unemployment in the communities.*** A comparison of Agricultural Extension Services with reduction in unemployment revealed that the mean score associated with respondents’ perception of the extent to which Edo ADP has influenced the reduction of unemployment in their communities was 3.0504 with a standard deviation of 1.037 and a standard error mean of 0.06585. When this was compared with a test value of 3, it resulted in a mean difference of 0.1653. A t-test for equality of means revealed that the resultant mean difference was not significant at the five per cent level since the significant p value of 0.445 is not less than 0.05 the assumed level of significance. Consequently, at the 95% confidence level, the agricultural extension services of Edo ADP perceived not to have significantly influenced the reduction in the level of unemployment in the communities (see Table 5).

***Respondents’ perception does not vary with location*.** A comparison of respondents’ perception with their location revealed that there was a significant difference between respondents’ perception and location since the computed F and associated significant probabilities were 7.451 and 0.015 (p < 0.05) respectively, thus indicating that at 99% confidence level, the effect of ADP is different across the locations (see Table 7). A post hoc test revealed that the impact of ADP in Egor local government was perceived not be significantly different from Ikpoba-Okha but significantly different from Oredo (see Table 9 and Table 9.1).

**Table 9.** Agricultural Extension Services and Location of Respondents.

Source of variation	Sum of Squares	df.	Mean Square	F	Sig.
Between Groups (LGAs)	7.451	2	3.725	4.259	0.015
Within Groups (LGAs)	214.291	245	0.875		
Total	221.742	247			

**Table 9.1.** Post Hock Tests (Homogeneous Subsets).

		Subset for α = 0.05	
Location of Respondents	N	1	2
Egor Local Government Area	90	2.8903	
Ikpoba Okha Local Government Area	76	3.1086	3.1086
Oredo Local Government Area 82	82		3.3064

***Respondents’ perception and sex.*** A comparison of respondents’ perception with sex revealed that the perception of male respondents was not significantly different from that of female respondents in all the hypotheses tested since none of the significant probabilities was less than 0.05, the assumed level of significance. The implication is that there is no significant relationship between respondents’ perception and sex (see Table 6). Thus respondents’ perception is not related to sex

***Extension services of Edo ADP are not related to crop/farm and infrastructural development as well as reduction in unemployment.*** Table 8 presents the regression analysis with extension services of ADP serving as the dependent variable while crop/farm development, infrastructural development and reduction in unemployment are the independent variables. The intention of this model is to establish the nature of the relationship between extension services of ADP and the explanatory variables. Here, the argument is based on the fact that if Y is directly proportional to X, then X is also directly proportional to Y. the results show that the adjusted R square is 0.526, thus indicating that the independent variables explain 52.6% of the variation in the dependent variable. Furthermore, crop/farm development and infrastructural development were significantly related to extension services of ADP. The reduction in unemployment was not significant. The ANOVA results show a calculated F statistic of 9.2125, with an asymptotic significant probability of 0.00, thus indicating that the test is significant at the one per cent level. Thus, at the 95% confidence level, we can conclude that the overall significance of the model is good.

Participant responses from the Agricultural Development Project survey https://dx.doi.org/10.5256/f1000research.16568.d225570
Copyright: © 2019 Inegbedion H et al. 2019 ${data-license-text}

### Discussion of findings

The findings show that agricultural extension services of ADP are perceived to have had significant impact on crop and farm development. The perceived impact of Agricultural Extension Services of Edo ADP on crop and farm development implies significant economic benefits to the farmers through enhancement in earnings from farming. However, the results do not indicate significant impact on reduction in unemployment and development of infrastructural facilities in the sampled communities. The results are consistent with the findings of Olujenyo (2006); Omonijo *et al.* (2014); Ugwu (2007) and Umeh *et al*. (2015). Also, the non-significance of agricultural extension services of ADP on infrastructural facilities is consistent with the findings of Adamu & Mohammed (2009). Furthermore, the results show that the impact of ADP on the local governments is not the same. In other words, the extent of impact of the ADP differs across the locations (local governments). In other words, the impact of the ADP is more in some locations than others. This is consistent with Enwelu *et al*. (2017). Lastly, the regression results show that the development of infrastructural facilities is perceived to be significantly related to agricultural extension services of the ADP but the t test for significance shows that agricultural extension services of the ADP has not had a significant impact on the development of infrastructural facilities. This may not be unconnected with the uneven impact of the ADP in the respective locations. This partial impact of ADP on the development of infrastructural facilities is a point of departure from previous studies that examined impact of ADP and declared the project non-significant without ascertaining the degree of non-significance of the project.

### A model of ADP performance

**Figure 2.**
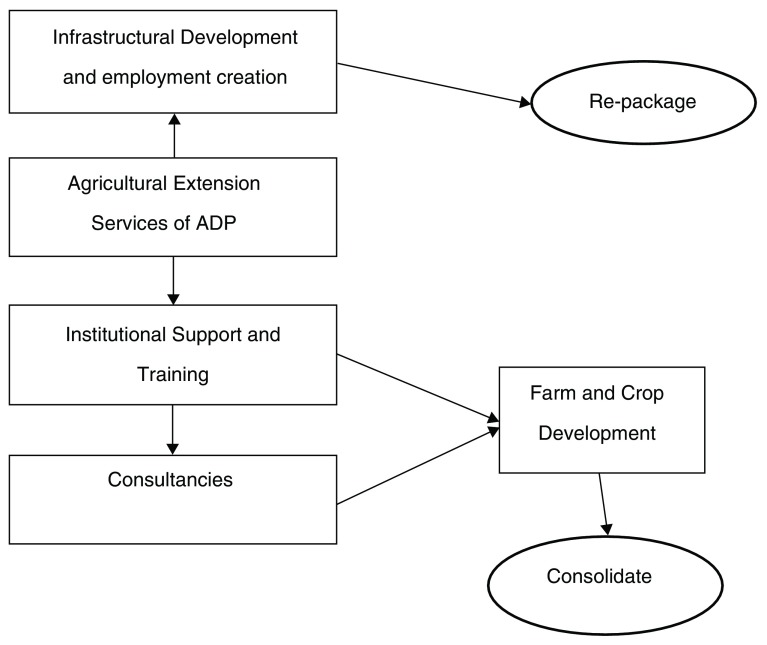
Suggested model of Agricultural Development Programme (ADP) performance in Edo State, Nigeria.

The proposed model shows that the agricultural extension programmes (institutional support and training and consultancies) of the ADP have been impactful in farm and crop development and so should be consolidated in these areas. But the programmes have not been impactful in infrastructural development and employment creation. If these goals are to be achieved, the programme should be repackaged (see Figure 2)

## Conclusions

The agricultural extension services of ADP have so far impacted crop and farm development significantly, but have not had significant impact on infrastructural development and reduction in rural unemployment. The non-significance of the ADP on development of infrastructural facilities is due to the uneven implementation of the programme across locations as revealed by the ANOVA test. This accounts for the conflict between the results of the t test and the least squares test. To this end, the extension services have been impactful but have not fully exerted the desired impact on the communities in Edo state. The pattern of intervention in some of the areas was significantly different others, which explains the disparity in impact across the locations (local governments) as some of the locations were more impacted by the others

This study has made significant contribution to agricultural research literature by updating previous studies on ADP in Nigeria. Furthermore, the study revealed that while crop and farm development was significantly impacted by the ADP across all locations, development of infrastructural facilities was impacted in some locations but not in others. The partial impact of ADP on the development of infrastructural facilities as a result of uneven implementation of the programme across locations is a major contribution of this study. The study also proposed a model to capture the impact of ADP on agricultural financing till date.

The study was not without limitations, which could constrain the generalizability of the results of this study. First, the inability of the researcher to obtain a completely random sample, owing to logistic concerns, especially transportation, in the rural areas was a limitation because randomness is a prerequisite for representativeness. Nevertheless, efforts were made to minimize this constraint through the use of alternative transport mediums like tricycles and motorcycles. Secondly, the reluctance of some sampled respondents to volunteer the desired information was a big challenge and could have further distorted the desired randomness in sample selection. The reluctance was tackled by adequate sensitization which eventually saw approximately 68% of the sample responding to the interview.

## Recommendations/suggestions for further studies

In view of the findings, the following recommendations are suggested: Firstly, concerned authorities should show adequate commitment and willingness to the success of the ADP by re-strategizing and reengineering the extension services with a view to making it more encompassing in order to yield the desired impact; secondly, concerted efforts should be made to use the ADP as a means of rejuvenating the agricultural sector to boost the self-reliance and self-sufficiency of the food production agenda of the federal government of Nigeria as well as the diversification of the nation’s resource base; lastly, there is the need to also redesign the scheme to address the shortcomings, which are currently threatening to truncate the programme. Future studies should attempt to increase the sample size used in this study in order to enhance the reliability of the findings consistent with the central limit theory.

## Data availability

F1000Research: Dataset 1. Participant responses from the Agricultural Development Project survey, https://doi.org/10.5256/f1000research.16568.d225570 Inegbedion *et al.* (2018).

## Supplementary material

Supplementary File 1: Study questionnaire

## References

[ref-1] Adamu MU Mohammed WA: The effect of agricultural development project (ADP) on the rural farmers in Adamawa state, Nigeria. *Asian J Agric Rural Dev.* 2009;2(3):405–410. Reference Source

[ref-2] Agbadudu AB: Statistics for business and the social sciences, Benin: Uri publishing company limited.2007.

[ref-3] Agbonifoh BA Yomere GO: Research methodology in the social sciences and Education, Benin City: Centrepiece Consultancy limited.2002.

[ref-4] Ammani AA Auta SJ Aliyu JA: Challenges to sustainability: Applying the problem tree analysis methodology to the ADP system in Nigeria. *J Agric Ext.* 2010;14(2):36–46. 10.4314/jae.v14i2.64122

[ref-31] Auta SJ Dafwang II: The agricultural development projects (ADPs) in Nigeria: Status and policy implication. *Res J Agric Biol Sci.* 2010;6(2):138–143. Reference Source

[ref-32] Chukwuemeka E Nzewi HN: An empirical study of World Bank agricultural development programme in Nigeria. *American Journal of Social and management Sciences.* 2011;2(1):176–187. 10.5251/ajsms.2011.2.1.176.187

[ref-6] Cochran WG: Sampling techniques.New York: John Wiley and Sons, Inc.1963 Reference Source

[ref-8] Enwelu IA Enwereuzor SO Asadu AA: Access and Use of Information and Communication Technologies by Extension Workers in Anambra State Agricultural Development Programme, Nigeria. *J Agric Ext.* 2017;21(2):152–161. 10.4314/jae.v21i2.13

[ref-23] Independent Evaluation Group: Agricultural developments in Nigeria.2009.

[ref-9] Inegbedion HE: Oil price hike and the Nigerian economy. Saarbrucken, Germany: Lap Lambert Academic Publishing. GmbH & Co. KG. ISBN-10: 3659117986.2012 Reference Source

[ref-10] Inegbedion HE Obadiaru ED: Modelling brand loyalty in the Nigerian telecommunications industry. *Journal of Strategic Marketing.* 2018 10.1080/0965254X.2018.1462842

[ref-24] Inegbedion HE Obadiaru DE Bello DV: Factors that influence consumers’ attitude towards internet buying in Nigeria. *Journal of Internet Commerce.* 2016;15(4):353–375. 10.1080/15332861.2016.1252646

[ref-11] Inegbedion H Obadiaru E Obasaju B: Dataset 1 in: Financing Agriculture in Nigeria through Agricultural Extension Services of Agricultural Development Programmes(ADPs). *F1000Research.* 2018 10.5256/f1000research.16568.d225570 PMC6556997 31231505

[ref-12] Naswem AA Ejembi SA: Reviving agricultural extension for effective transition from subsistence to commercial agriculture in Nigeria. *J Rural Soc Sci.* 2017;32(1):3–20. Reference Source

[ref-13] Nchuchuwe FF Adejuwon KD: The Challenges of agriculture and rural development in Africa: The case of Nigeria. *International Journal of Academic Research in Progressive Education and Development.* 2012;1(3):45–61. Reference Source

[ref-14] Nwachukwu IN Eze CI: Impact of selected rural development programmes on poverty alleviation in Ikuano Local Government Area, Abia state Nigeria. *Afr J Food Agric Nutr Dev.*Kenya.2007;7(5):1–17. Reference Source

[ref-16] Olujenyo FO: Impact of Agricultural Development Programme (ADP) on the Quality of Social Existence of Rural Dwellers in Developing Economies The Ondo State (Nigeria) Agricultural Development Programme Experience. *International Journal of Rural Management.* 2006;2(2):213–226. 10.1177/097300520600200205

[ref-33] Okuokenye GF Okoedo-Okojie DU: Evaluation of extension agents’ commitment to the agricultural loans and inputs supply programme on special rice production in Delta state, Nigeria. *Journal of Applied Science and Environmental Management.* 2014;18(2):327–335. 10.4314/jasem.v18i2.25

[ref-17] Omonijo DO Toluwase SOW Oludayo OA: Impacts of Agricultural Development Programme(ADP) on Rural Dwellers in Nigeria: A Study of Isan-Ekiti. *International Research Journal of Finance and Economics.* 2014;128:41–55. Reference Source

[ref-18] Tunji Titilola ST: Environment and sustainable agricultural development in Nigeria(online).2001 Reference Source

[ref-21] Ugwu DS: Contributions of agricultural development programmes (ADPs) to rural livelihood and food security in Nigeria. *Agricultural Journal.* 2007;2(4):503–510. Reference Source

[ref-22] Umeh OJ Ekumankama OO Nwachukwu I: Comparative performance evaluation of the Agricultural development programmes of Abia and Enugu states, Nigeria. *J Agric Ext.* 2015;19(2):108–114. 10.4314/jae.v19i2.9

