# Supplementary material for: Financing Agriculture in Nigeria through Agricultural Extension Services of Agricultural Development Programmes (ADPs)
Source: F1000Res. 2019 May 30;7:1833. Originally published 2018 Nov 21. [Version 3] doi: 10.12688/f1000research.16568.3 (PMC6556997; doi:10.12688/f1000research.16568.3)
Supplement: Supplementary file 2 [file f1000research-7-21336-s0001.tgz › 5cde479e-e0a7-4152-accf-95a050498e74_Supplementary_file_1.docx]

**Questionnaire**

**SECTION A**

**Instruction:** Mark X in the box as appropriate **[X]**

1. Age category in years**:** Under 30 [ ] 31 – 40 [ ] 41 – 50 [ ] 51 and above [ ]

2. Sex: Male [ ] Female [ ]

3 Marital status: Married [ ] Single [ ] Divorced [ ] Widowed [ ]

4. Local Government Area: Oredo [ ] Egor [ ] Ikpoba Okha [ ]

**INSTRUCTION**: Please indicate the extent to which you agree with the following items by writing 1, 2, 3, 4 or 5 in the response box.

Note: Strongly Agree = 5; Agree = 4; No View = 3; Disagree = 2; and Strongly Disagree =1

| **S/N** | **Construct/Questions** | **Response** |
| --- | --- | --- |
|  | **Farm and Crop Development** |  |
| Q1 | Agricultural extension services of Edo ADP have impacted food crop development in my community |  |
| Q2 | Agricultural extension services of Edo ADP have impacted cash crop development in my community |  |
| Q3 | Agricultural extension services of Edo ADP have impacted farm development in my community |  |
|  | **Infrastructural Development** |  |
| Q4 | Agricultural extension services of Edo ADP have enhanced the provision of water my community |  |
| Q5 | Agricultural extension services of Edo ADP have enhanced the provision of access roads my community |  |
| Q6 | Agricultural extension services of Edo ADP have enhanced the provision of basic amenities my community |  |
|  | **Reduction of Unemployment** |  |
| Q7 | Agricultural extension services of Edo ADP have increased job opportunities in my community |  |
| Q8 | Agricultural extension services of Edo ADP have reduced the level of unemployment my community |  |
