# Supplementary material for: Financing Agriculture in Nigeria through Agricultural Extension Services of Agricultural Development Programmes (ADPs)
Source: F1000Res. 2019 May 30;7:1833. Originally published 2018 Nov 21. [Version 3] doi: 10.12688/f1000research.16568.3 (PMC6556997; doi:10.12688/f1000research.16568.3)
Supplement: Peer review history

# Peer review: Reviewer response for version 3

**DOI:** 10.5256/f1000research.21336.r49277

**Published:** 2019-06-03

**Authors:** David L. Sjoquist

**Affiliations:** 1 Department of Economics, Andrew Young School of Policy Studies, Georgia State University, Atlanta, GA, USA

The authors have adequately addressed my concerns with the previous version.

I have read this submission. I believe that I have an appropriate level of expertise to confirm that it is of an acceptable scientific standard.

# Peer review: Reviewer response for version 2

**DOI:** 10.5256/f1000research.21073.r48390

**Published:** 2019-05-23

**Authors:** David L. Sjoquist

**Affiliations:** 1 Department of Economics, Andrew Young School of Policy Studies, Georgia State University, Atlanta, GA, USA

There is a mismatch between the discussion and the table number that is referenced in the text. For example, the discussion of development of infrastructure cites table 5, but the analysis is in Table 4. Likewise, in the text Table 6 should be Table 5.

 The standard deviation and standard error for crop development are 3.153 and 0.069 in the text, but 3.1694 and 0.0669 in Table 3. Which are the correct numbers?

 Table 4 is referenced in the discussion of farm development, but Table 4 concerns infrastructure development. There is no table that reports results for farm development.

 Tables 6 and 7 are not discussed in the text, so it is unclear what they mean.

I have read this submission. I believe that I have an appropriate level of expertise to confirm that it is of an acceptable scientific standard, however I have significant reservations, as outlined above.

# Response

**Published:** 2019-05-25

**Authors:** Henry INEGBEDION

**Affiliations:** Landmark University, Nigeria

We apologise for the mismatch between the discussion of findings and some of the table numbers. We have made adequate corrections by situating the table numbers correctly in the text. We appreciate the peer reviewers for their contributions to the enhancement of the quality of this article.

# Peer review: Reviewer response for version 2

**DOI:** 10.5256/f1000research.21073.r48391

**Published:** 2019-05-20

**Authors:** Phillip Alege

**Affiliations:** 1 Covenant University (CU), Ota, Nigeria

I have no further comments to make.

I have read this submission. I believe that I have an appropriate level of expertise to confirm that it is of an acceptable scientific standard.

# Peer review: Reviewer response for version 1

**DOI:** 10.5256/f1000research.18109.r47572

**Published:** 2019-04-29

**Authors:** David L. Sjoquist

**Affiliations:** 1 Department of Economics, Andrew Young School of Policy Studies, Georgia State University, Atlanta, GA, USA

The literature review is more like an annotated biography. I would have liked to have seen some discussion of the limitations of the existing research. There is no discussion of how the current research adds to or improves upon the existing literature.

 While the survey questions are available, it would be very helpful if the questions were made part of the paper. Furthermore, a table of the responses would be helpful for the reader. The existing table only provides the mean response, but the distribution of the responses would be very helpful in understanding the results.

 A minor point, but the first three and second three tables could each be combined into one table, saving some space.

 The table numbers referred to in the text do not match the table numbers. Tables 5 and Table 6 both refer to educational attainment, while the text discusses location and refers to table 5. There is a reference to Table 5.1, but there is no table 5.1 in the paper.

 The table titles and hypotheses statements could be improved; the current title makes it seem that the analysis is pitting the ADP program against (“versus”), for example, crop development.   

 The survey asks multiple questions on some issues, but the authors do not explain what question or questions are used to calculate the means reported in tables.

 The authors misstate the what the tables show. For example, they state that “…ADP have significantly affected…” What the survey can tell us, is that these services are **perceived** to have affected, say farm development, which is substantially different from knowing that the services actually affected, say farm development.

 The authors note when the effects are statistically significant. But, it would be important to note whether the effects are economically significant.

 The paper suggests some policy changes. However, the research reported in the paper does not provide support for (or against) the various policy recommendation. For example, the authors state that the authorities should re-strategize and reengineer the extension services. But there are no questions in the survey that suggest that such changes are necessary or would improve the services.

I have read this submission. I believe that I have an appropriate level of expertise to confirm that it is of an acceptable scientific standard, however I have significant reservations, as outlined above.

# Response

**Published:** 2019-05-02

**Authors:** Henry INEGBEDION

**Affiliations:** Landmark University, Nigeria

The literature review is more like an annotated biography. I would have liked to have seen some discussion of the limitations of the existing research. There is no discussion of how the current research adds to or improves upon the existing

**Response:** **Additional Empirical studies, find below**

Auta and Dafwang (2010) investigated the status and policy of ADPs in Nigeria. they found that over sixty three percent of the ADPs had weak or very weak funding status while over twenty two percent had good to excellent status. Furthermore, most of the ADPs had reduced their extension workers in recent times due to poor funding

Chukwuemeka and Nzewi (2011) investigated World Bank sponsored Agricultural Development Project to find out the extent to which the programme had achieved set objectives. Survey and questionnaire served as research design and instrument. Findings showed that beneficiaries were excluded from design, planning and implementation of project; a development that was perceived as undesirable. Besides, political considerations, rather than expertise and professionalism was found to characterise the recruitment of extension staff; and the joint financiers (World Bank, Federal and State governments of Nigeria) were not fulfilling their financial obligations as at when due.

Okuokenye and Okoedo-Okojie (2014) investigated activities of extension agents on agricultural loans and inputs supply programme participant farmers’ rice output/income. Questionnaire served as the instrument of data collection from a sample of 60 randomly selected extension farmers and 80 participant and 80 non-participant farmers’. Results showed that the extension agents made significant impact on output and income. The major constraints to the implementation of the programme were found to be restricted coverage of farms and wrong selection of participants

Naswem and Ejembi (2017) historically examined the extent to which the agricultural extension programme has enhanced transition from subsistence to commercial agriculture in Nigeria. they observed that the programme had made some significant impact but was currently constrained by dwindling oil revenues and, most importantly, the absence of a coherent extension policy

**Comment:**  The literature review is more like an annotated biography. I would have liked to have seen some discussion of the limitations of the existing research. There is no discussion of how the current research adds to or improves upon the existing literature.

**Response:**

2.2.1. *Research Gap*

 The empirical review reveals that adequate empirical literature abound on agricultural financing through the extension services of ADP in Nigeria. Most of the studies agree that the ADPs have made significant impact on agricultural production in Nigeria, especially in the a areas of increased agricultural output and income as well as improved rural livelihood (Ammani, Auta & Aliyu, 2010; Olujenyo, 2006; Ugwu, 2007; Adamu & Mohammed, 2009; Omonijo, Toluwase, Oludayo & Uche, 2014; and Okuokenye & Okoedo-Okojie, 2014). However, not all the objectives of the programme have been successful. Specifically, the provision of credit facilities (Omonijo, Toluwase, Oludayo & Uche (2014) and infrastructural developments (Adamu & Mohammed, 2009). Furthermore, despite the perceived positive impact of the ADP in agricultural outputs and income, findings also indicate that there are challenges currently being faced by the programme in a significant number of the states where it is being implemented. These challenges could erode the credibility and “Goings Concern” of the implementation of the project if urgent steps are not taken to mitigate the challenges. The major challenges include inadequate funding, mainly as a result of the inability of critical stakeholders (World Bank, federal government and State governments) to fulfill their financial obligations to the programme as and when due (Ugwu, 2007; Ammani, Auta & Aliyu, 2010; Chukwuemeka & Nzewi, 2011) Omonijo, Toluwase, Oludayo & Uche, 2014; Auta & Dafwang, 2010; and Okuokenye & Okoedo-Okojie, 2014) as well as politicization of the selection of participants in the implementation of the programme by government (Ugwu, 2007; Chukwuemeka & Nzewi, 2011; and Okuokenye & Okoedo-Okojie, 2014).

In view of the foregoing, the need to re-awaken the interest of stakeholders on the actualization of the objectives of the ADP becomes very important. Besides, the perceived insignificant impact on infrastructural development should attract the interest for stakeholders for adequate intervention. The constraints to the continued implementation of the scheme also raise some questions to stakeholders. This study attempts to fill these gaps

**Comment**

While the survey questions are available, it would be very helpful if the questions were made part of the paper. Furthermore, a table of the responses would be helpful for the reader.

**Response**

**Table A          Demographic Distribution of respondents**

            Age                             Category                                 F                      %

Under 30 Years                       12                    4.8

31-40 Years                             75                    30.2

41-50 Years                             105                  42.3

50 Years and above                56                    22.6

Total                                        248                  100

            Sex                              Male                                        169                  68.1

                                                Female                                     31.9                 31.9

                                                Total                                        248                  100

            Marital Status              Married                                   191                  77

                                                Single                                      38                    15.3

                                                Widowed                                15                    6

                                                Divorced                                 4                      1.6      

                                                Total                                        248                  100

            Highest Education      SSCE                                      113                  45.6

                                                ND/NCE                                 82                    33.1

                                                HND/First Degree                  51                    20.6

                                                Higher Degree                         2                      8

                                                Total                                        248                  100     

            Local Govt. Area        Oredo                                      82                    33.1                

                                                Egor                                        90                    36.3

                                                Ikpoba Okha                           76                    30.6

                                                Total                                        248                  100

**Table B           Response to Items on Research Problem**

            Variable                                               Item     SD       D         NV      A         SA

1. Farm and Crop Development                          Q1       10        70        58        62        28       

                                                                                    Q2       14        75        54        79        26

                                                                                     Q3       9          72        61        81        25

1. Infrastructural Development                             Q4       20        76        53        75        24

                                                                                     Q5       14        73        57        79        24

                                                                                     Q6       21        71        57        70        29

1. Reduction in Unemployment                             Q7       17        75        55        78        23

                                                                                     Q8       18        24        57        78        21

**Comment**

- **Are the conclusions drawn adequately supported by the results?**

No

**Response**

An additional conclusion not initially captured is presented below:

The pattern of intervention in some of the areas was significantly different fom others, thus the impact of the extension programmes of ADP are not uniform across locations but vary from location to location

# Peer review: Reviewer response for version 1

**DOI:** 10.5256/f1000research.18109.r43240

**Published:** 2019-03-15

**Authors:** Phillip Alege

**Affiliations:** 1 Covenant University (CU), Ota, Nigeria

The literature review is not adequate. The literature review lacks depth and spread.

 General Comments:

 The author should provide enough review of studies in similar areas covering the theoretical explanations. Also, the review should be considered over a period of time. That is, the review should focus on old and recent studies in the subject area so as to clearly situate the gaps to be filled in the literature.

 FADAMA

 The author provide the full meaning of the acronym when it is mentioned for the first time.

 Grammatical Errors

 The authors should cross-check the document for errors. Example: author wrote "where"  instead of "were"

 Spelling Errors of Authors Cited

 The authors should check the correct name of an author on page 6. The author wrote "Cochranor" instead of "Cochrane"

 Mathematical Errors

 The authors should rewrite the mathematical formula correctly in Page 6, Paragraph 3

 Additional Information: Hypotheses statements

 The statements of hypotheses in the work are not correctly stated.

 For example, Hypothesis One states that: Agricultural Extension Services of Edo Agricultural Development Programme (ADP) vs. crop development

 However, the statement is not testable. Hypothesis should be stated in the null and alternative form to be testable.

I have read this submission. I believe that I have an appropriate level of expertise to confirm that it is of an acceptable scientific standard, however I have significant reservations, as outlined above.

# Response

**Published:** 2019-03-15

**Authors:** Henry INEGBEDION

**Affiliations:** Landmark University, Nigeria

We accept responsibility for the editing (grammatical errors) and promise to review them. we may also do a minor update of the literature in line with the reviewer's comments. However, we do not think it is compulsory to state the null and alternative hypotheses to confirm statistical testability. often times, it is the null hypothesis that is tested in a study, not the null and alternative. Once the null hypothesis is formulated, the alternative hypothesis is implied, as the null hypothesis often denies the existence of the relationship between two or more variables.

# Response

**Published:** 2019-05-02

**Authors:** Henry INEGBEDION

**Affiliations:** Landmark University, Nigeria

Comment:   The literature review is not adequate. The literature review lacks depth and spread.

**Response:** **Additional Empirical studies, find below**

Auta and Dafwang (2010) investigated the status and policy of ADPs in Nigeria. they found that over sixty three percent of the ADPs had weak or very weak funding status while over twenty two percent had good to excellent status. Furthermore, most of the ADPs had reduced their extension workers in recent times due to poor funding

Chukwuemeka and Nzewi (2011) investigated World Bank sponsored Agricultural Development Project to find out the extent to which the programme had achieved set objectives. Survey and questionnaire served as research design and instrument. Findings showed that beneficiaries were excluded from design, planning and implementation of project; a development that was perceived as undesirable. Besides, political considerations, rather than expertise and professionalism was found to characterise the recruitment of extension staff; and the joint financiers (World Bank, Federal and State governments of Nigeria) were not fulfilling their financial obligations as at when due.

Okuokenye and Okoedo-Okojie (2014) investigated activities of extension agents on agricultural loans and inputs supply programme participant farmers’ rice output/income. Questionnaire served as the instrument of data collection from a sample of 60 randomly selected extension farmers and 80 participant and 80 non-participant farmers’. Results showed that the extension agents made significant impact on output and income. The major constraints to the implementation of the programme were found to be restricted coverage of farms and wrong selection of participants

Naswem and Ejembi (2017) historically examined the extent to which the agricultural extension programme has enhanced transition from subsistence to commercial agriculture in Nigeria. they observed that the programme had made some significant impact but was currently constrained by dwindling oil revenues and, most importantly, the absence of a coherent extension policy

**Comment:**  The literature review is more like an annotated biography. I would have liked to have seen some discussion of the limitations of the existing research. There is no discussion of how the current research adds to or improves upon the existing literature.

**Response:**

2.2.1. *Research Gap*

 The empirical review reveals that adequate empirical literature abound on agricultural financing through the extension services of ADP in Nigeria. Most of the studies agree that the ADPs have made significant impact on agricultural production in Nigeria, especially in the a areas of increased agricultural output and income as well as improved rural livelihood (Ammani, Auta & Aliyu, 2010; Olujenyo, 2006; Ugwu, 2007; Adamu & Mohammed, 2009; Omonijo, Toluwase, Oludayo & Uche, 2014; and Okuokenye & Okoedo-Okojie, 2014). However, not all the objectives of the programme have been successful. Specifically, the provision of credit facilities (Omonijo, Toluwase, Oludayo & Uche (2014) and infrastructural developments (Adamu & Mohammed, 2009). Furthermore, despite the perceived positive impact of the ADP in agricultural outputs and income, findings also indicate that there are challenges currently being faced by the programme in a significant number of the states where it is being implemented. These challenges could erode the credibility and “Goings Concern” of the implementation of the project if urgent steps are not taken to mitigate the challenges. The major challenges include inadequate funding, mainly as a result of the inability of critical stakeholders (World Bank, federal government and State governments) to fulfill their financial obligations to the programme as and when due (Ugwu, 2007; Ammani, Auta & Aliyu, 2010; Chukwuemeka & Nzewi, 2011) Omonijo, Toluwase, Oludayo & Uche, 2014; Auta & Dafwang, 2010; and Okuokenye & Okoedo-Okojie, 2014) as well as politicization of the selection of participants in the implementation of the programme by government (Ugwu, 2007; Chukwuemeka & Nzewi, 2011; and Okuokenye & Okoedo-Okojie, 2014).

In view of the foregoing, the need to re-awaken the interest of stakeholders on the actualization of the objectives of the ADP becomes very important. Besides, the perceived insignificant impact on infrastructural development should attract the interest for stakeholders for adequate intervention. The constraints to the continued implementation of the scheme also raise some questions to stakeholders. This study attempts to fill these gaps

**Comment**

Grammatical Errors

1. The authors should cross-check the document for errors. Example: author wrote "where"  instead of "were" Spelling Errors of Authors Cited: The authors should check the correct name of an author on page 6.
2.  The author wrote "Cochranor" instead of "Cochrane"

**Response**

1. This has been corrected on page 4 in the following paragraph: The FADAMA programme of the ADP, which began in the north has also taken off in the south and is currently undertaken by Edo ADP. FADAMA involves interventionist programmes, using relevant methodologies in areas where water is close to the surface of the earth. The frequency of . ..
2.  This has been corrected in the paragraph:      The value for Z is obtained from the normal distribution table, from the area under the normal curve (Cochrane, 1963; and Agbadudu, 2007).

**Comment:**

Additional Information: Hypotheses statements

The statements of hypotheses in the work are not correctly stated.

For example, Hypothesis One states that: Agricultural Extension Services of Edo Agricultural Development Programme (ADP) vs. crop development

**Response**

**The hypotheses have been reformulate to reflect this correction**

**Hypotheses**

1. Agricultural extension services of Edo ADP have not significantly affected crop development in the communities
2. Agricultural extension services of Edo ADP have not significantly affected farm development in the communities
3. Agricultural extension services of Edo ADP have not significantly affected the development of infrastructural facilities in the communities
4. Agricultural extension services of Edo ADP have not significantly influenced reduction in the level of unemployment in the communities

 
**Comment:      **Mathematical Errors

The authors should rewrite the mathematical formula correctly in Page 6, Paragraph 3

Response

The correct formula is presented below:

n = ^,.^

# Response

**Published:** 2019-05-10

**Authors:** Henry INEGBEDION

**Affiliations:** Landmark University, Nigeria

This revised version has taken care of virtually all the errors that were identified by the reviewers. We have also revised the literature in line with the reviewer's comments and included a section on gap analysis. The hypotheses have now been stated in the null form to address the concern raised by the first reviewer. We have no competing interests to disclose.
